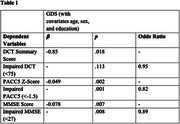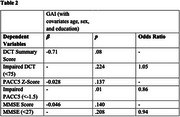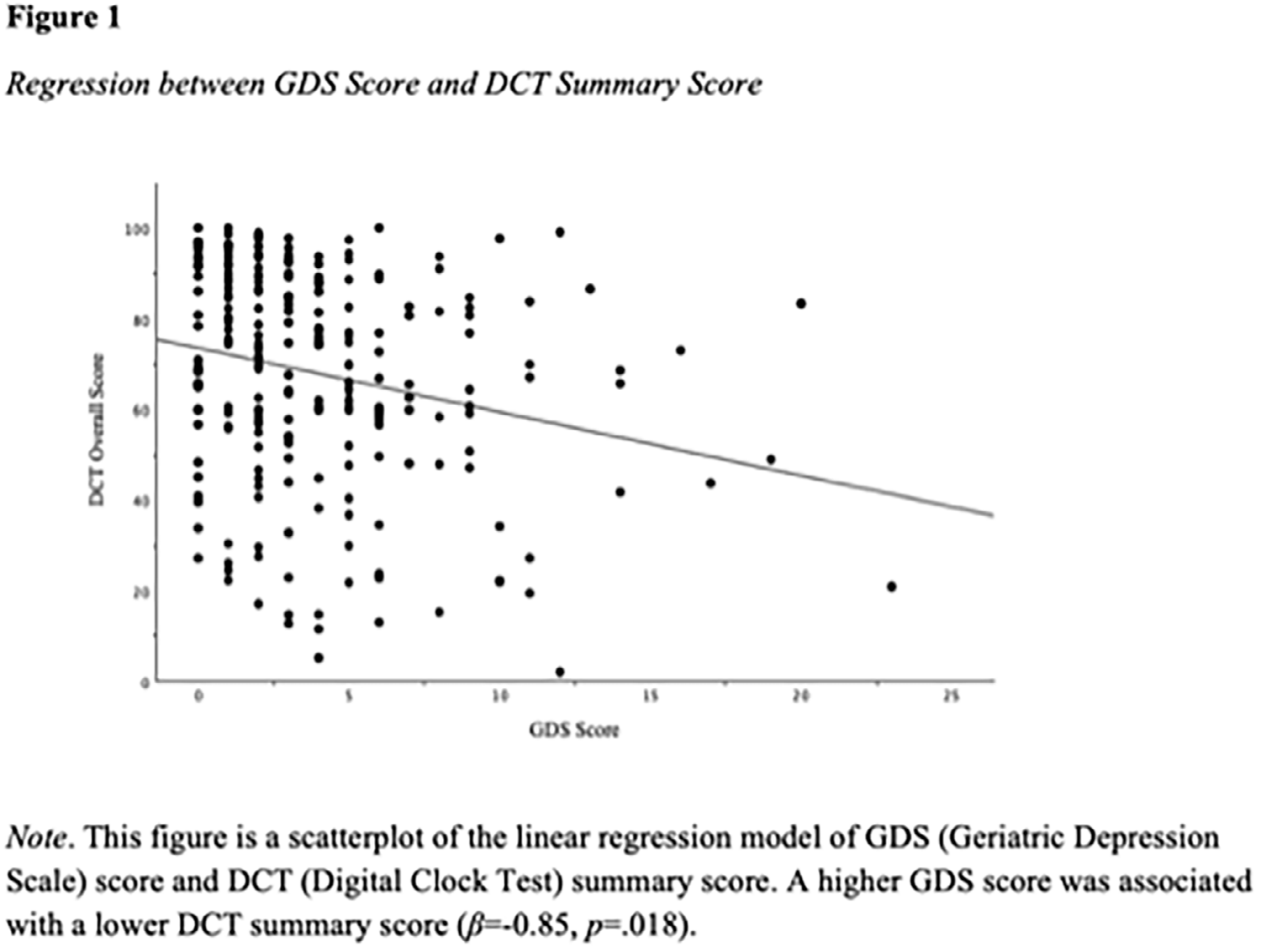# Does Mood Matter: The impact of affective symptoms on the Digital Clock Test scores, a portable and sensitive screen for cognitive impairment

**DOI:** 10.1002/alz70857_106486

**Published:** 2025-12-26

**Authors:** Abigail LaCasse, Natalie R Scher, Jessie Fanglu Fu, Talia L. Robinson, Dorene M. Rentz, Rebecca E. Amariglio, Kathryn V Papp, Yakeel T. Quiroz, Deborah Blacker, Reisa A. Sperling, Keith A. Johnson, Gad A. Marshall, Catherine E Munro, Jennifer R. Gatchel

**Affiliations:** ^1^ William James College, Newton, MA, USA; ^2^ Brigham and Women's Hospital, Boston, MA, USA; ^3^ Massachusetts General Hospital, Boston, MA, USA; ^4^ Athinoula A Martinos Center for Biomedical Imaging, Massachusetts General Hospital, Harvard Medical School, Charlestown, MA, USA; ^5^ Massachusetts General Hospital, Harvard Medical School, Boston, MA, USA; ^6^ Massachusetts General Hospital, Harvard Medical School, Department of Neurology, Boston, MA, USA; ^7^ Center for Alzheimer's Research and Treatment, Brigham and Women's Hospital, Harvard Medical School, Boston, MA, USA; ^8^ Center for Alzheimer's Research and Treatment, Department of Neurology, Brigham and Women's Hospital, Harvard Medical School, Boston, MA, USA; ^9^ Massachusetts General Hospital, Department of Neurology, Harvard Medical School, Boston, MA, USA; ^10^ Department of Psychological & Brain Sciences, Boston University, Boston, MA, USA; ^11^ Grupo de Neurociencias de Antioquia, University of Antioquia, Colombia, Medellín, Antioquia, Colombia; ^12^ Harvard University, Boston, MA, USA; ^13^ Department of Epidemiology, Harvard T. H. Chan School of Public Health, Boston, MA, USA; ^14^ Center for Alzheimer's Research and Treatment, Brigham and Women's Hospital/Harvard Medical School, Boston, MA, USA; ^15^ Department of Neurology, Massachusetts General Hospital, Harvard Medical School, Boston, MA, USA; ^16^ Center for Alzheimer's Research and Treatment, Brigham and Women's Hospital, Harvard Medical School, Boston, MA, USA; ^17^ Gordon Center for Medical Imaging, Department of Radiology, Division of Molecular Imaging and Nuclear Medicine, Massachusetts General Hospital, Harvard Medical School, Boston, MA, USA; ^18^ Center for Alzheimer Research and Treatment, Department of Neurology, Brigham and Women's Hospital, Boston, MA, USA; ^19^ Harvard Medical School, Boston, MA, USA; ^20^ Brigham and Women's Hospital/Massachusetts General Hospital, Boston, MA, USA; ^21^ Department of Psychiatry, Massachusetts General Hospital, Harvard Medical School, Boston, MA, USA; ^22^ McLean Hospital, Belmont, MA, USA

## Abstract

**Background:**

The Digital Clock Test (DCT) is a novel, computerized version of the clock drawing test (CDT). It has been shown to discriminate well between mild cognitive impairment and Alzheimer's disease (AD) dementia and has been associated with AD biomarkers (i.e., amyloid, tau), similar to widely used screening batteries such as the Preclinical Alzheimer's Cognitive Composite (PACC5) and Mini Mental Status Exam (MMSE). As mood symptoms (e.g., anxiety, depression) are common in older adults and can impact cognition, it is important to determine whether such symptoms impact scores on the DCT relative to other measures/batteries. We sought to examine whether mood symptoms are associated with lower DCT scores and/or impaired performance and compare relationships to the MMSE and PACC5.

**Method:**

All participants were older adults (*n* = 241, mean age=77.60, 80.10% cognitively normal) enrolled in the Harvard Aging Brain Study. Participants completed neuropsychological assessments (i.e., DCT, PACC5, MMSE; lower scores=worse performance) alongside self‐report questionnaires: the 30‐item Geriatric Depression Scale (GDS) and the 20‐item Geriatric Anxiety Inventory (GAI) (higher scores = greater symptoms for both measures). Separate cross‐sectional linear regression models assessed whether GDS/GAI score predicted DCT, MMSE, and PACC5 scores. Separate ordinal regression models were used to determine whether GDS/GAI score predicted impaired DCT (<75), MMSE (<27), and PACC5 (<‐1.5) scores. Analyses controlled for age, sex, and education.

**Result:**

Higher GDS scores, but not GAI scores, were associated with lower scores on all cognitive measures (Figure 1). Higher GDS scores were also associated with increased odds of having impaired MMSE and PACC5 scores, but not impaired DCT scores (Table 1). Additionally, higher GAI scores were significantly associated with increased odds of an impaired PACC5 score, but not impaired MMSE or DCT scores (Table 2).

**Conclusion:**

In older adults, greater depressive symptoms were associated with worse performance on both the DCT and standard cognitive screens (MMSE, PACC5). In contrast, both depressive and anxiety symptoms were associated with “impaired” scores on PACC5 and/or MMSE, but not on the DCT. This could suggest that the DCT is a sensitive cognitive screen for AD and related dementias in clinical settings where patients present with co‐occurring mood disturbances.